# Pediculosis humanus capitis Prevalence as a Health Problem in Girl’s Elementary Schools, Southwest of Iran (2017-2018)

**Published:** 2019-06-17

**Authors:** Hoda Ghofleh Maramazi, Mona Sharififard, Elham Jahanifard, Elham Maraghi, Mohammad Mahmoodi Sourestani, Amal Saki Malehi, Sima Rasaei

**Affiliations:** ^1^Department of Medical Entomology and Vector Control, School of Public Health, Ahvaz Jundishapur University of Medical Sciences, Ahvaz, Iran; ^2^Department of Biostatistics and Epidemiology, School of Public Health, Ahvaz Jundishapur University of Medical Sciences, Ahvaz, Iran; ^3^Department of Horticultural Science, Faculty of Agriculture, Shahid Chamran University of Ahvaz, Ahvaz, Iran; ^4^Department of Dermatology, School of Medicine, Ahvaz Jundishapur University of Medical Sciences, Ahvaz, Iran

**Keywords:** Lice infestation, Pediculosis, Head lice

## Abstract

**Background:** Head lice as obligated ectoparasite is a public health concern. We aimed to determine the prevalence of *Pediculus humanus * capitis as public health concern among girl’s primary school in southwest of Iran.

**Study design:** A cross-sectional study.

**Methods:** This study was conducted in Karoon County, south-west of Khuzestan Province in Iran in 2017-2018. Totally, 851 students were interviewed randomly, examined by a medical entomologist and completed a questionnaire containing 18 questions based on individual, social, economic, cultural and health information.

**Results** were presented as prevalence and percentages for qualitative variables and also, the data were analyzed by univariate logistic and multivariate regression models. Results: About 199 (23.38%) girls were infected by head lice. Univariate logistic regression indicated that the prevalence of pediculosis was directly associated with the grade of education, father’s job, shared personal hygiene products, number of combing, having permanent head cover at home, infection in other members of the family and previous infection. Multivariate logistic regressions for predicting of head lice infection in girl students were reported permanent head cover at home (OR: 1.399, 95% CI: 0.934, 2.097, *P*=0.104), grade of education (OR: 1.948, 95% CI: 1.307, 2.905, *P*=0.001), father’s job (OR: 2.385, 95% CI: 1.518, 3.750, *P*<0.001), shared personal hygiene products (OR: 1.817, 95% CI: 1.224, 2.698, *P*=0.003) and using hair oil (OR: 1.904, 95% CI: 1.279, 2.836, *P*=0.002) had significant relation with head lice infestation

**Conclusion:** Head lice remind as serious health problem in Karoon County, southwest of Iran. Due to high infestation, periodic screening of the student is recommended for early detection and treatment.

## Introduction

Lice as obligated ectoparasites have a long association with the human host and indicate a co-evolution between hosts and parasites^1, 2^. Overall, humans have been infested by lice and they spread according to human migrations out of Africa. The oldest head lice were reported on 8000-year-old fossil man in Brazil^3^.

Blood-sucking lice belong to the suborder of Anoplura and Pediculidae family is a wingless insect that feeds on sebaceous secretions and body fluids ^4, 5^. Nowadays, humans are the host of three lice species, including the head louse, the body louse and the pubic louse^6, 7^. Severe infection with the three species of lice leads to pediculosis^7^. Human head lice divided into A, B and C clad^3^. Furthermore, the separation of clade A happened into species (*Pediculus humanus* and *P. capitis*) or subspecies (*Pediculus humanus capitis* and *P. humanus humanus*) regarding mitochondrial DNA studies since the late 1970s^8^. Head and body lice have distinct differences in average size, color, behavioral and physiological adaptations, antennae forms and indentations between abdomen segments. *P. humanus capitis* do not carry diseases, but body lice are the vector of diseases like louse-born typhus and trench fever^7^. A head louse is a public health concern in the world. The prevalence of the insect has been reported in the range of 0.48% to 37% in Europe^9^.

Head louse is a contagious infection that often feeds on children in a range of 3 to 12 yr, but it can spread to the rest of the family^10^. The ectoparasite is mostly observed in crowded areas with close personal contact including schools, kindergarten, nurseries, sports, playgrounds, camps and prisons^11^. Head lice are transmitted directly from person to person or indirectly through common use of the comb, brush, pillow and cloths^12^. All growth stages of head lice, including nits, nymphs, and adults are mostly observed near the neckline at the behind of the head and back of the ears^7^.

Various factors like parents’ education and job, family size, presence of the bathroom at home, frequency of bathing per week and shared personal hygiene products effects on head lice infestations^13^. The scalp itchy is already due to lice bite, saliva, and faces^14^. The other symptoms are depression, insomnia, fatigue, educational failure, mental disorder, decreases the social stigma and allergic reactions ^5, 15^. The majority of cases of infestations are unreported due to social stigma^16^.

The prevalence of head lice was reported from 0.48% in Isfahan Province in the center of Iran to 27% in Sistan and Baluchistan in the southeast of the country^17^. The variation of head lice infestation rate is maybe due to lice resistance to treatment, socioeconomic status, poor healthcare, hair washing habits, family size, low knowledge about pediculosis, perception of head lice as a health problem, public culture, spatiotemporal distribution of hosts and lack of personal health^18^.

Regarding the cultural, social, health and economic status of Karoon County, as well as the lack of information about the prevalence of head lice infestation in the Khuzestan Province, the goal of this investigation was to determine the prevalence of *P. humanus capitis* as public health concern among girl’s primary schools in this county in southwest of Iran during 2017-2018.

## Methods

### Study area

**Figure 1:**
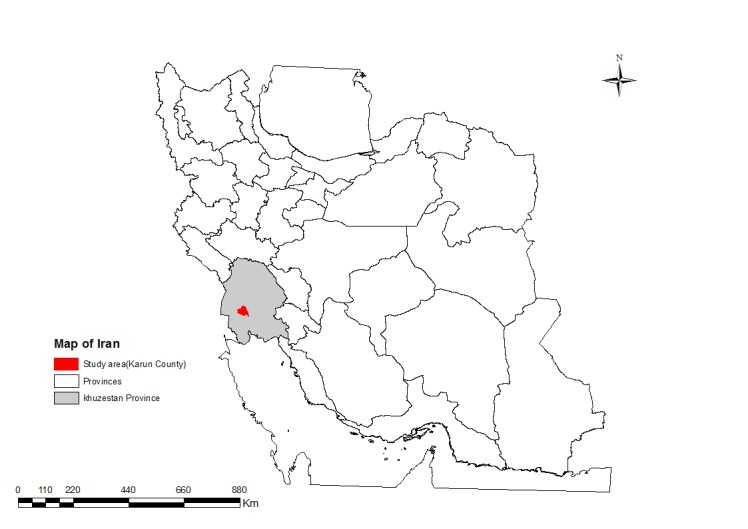
Study area, Karoon County, Khuzestan Province, southern Iran

Karoon County (30°26'N, 48°10'E) situated in south-west of Khuzestan Province in Iran (Figure 1). The county is divided into two districts including the Central and Soveyseh Districts. Kut-e Abdullah is the center of the county. The county has a warm, humid climate that temperatures reach above 50 °C during the warm summer season.

### Study design

This cross-sectional study was conducted during 2017-2018 in Karoon County, Iran. Inclusion criteria were sex (girl students), the grade of education, and governmental schools. An exclusion criterion was defined as a disagreement with parents. A questionnaire containing 18 questions based on an individual, social, economic, cultural and health information like location, grade of education (in educational system of Iran County, primary schools have six levels, entitled as "grade": grade I (7 yr old students), grade II (8 yr old students), grade III (9 yr old students), grade IV (10 yr old students), grade V (11 yr old students) and grade VI (12 yr old students), parent’s job, the number of students in class, family size, shared personal hygiene products (hairbrush, comb, towel, pillow, bed, hat), number of combing, Health care teacher at school, number of bathing per week, having itching, dandruff and head cover, hair type, using hair oil, background of family infestation, previous infestation and treatment type.

This project confirmed in the Ethical Committee of Ahvaz Jundishapur University of Medical Sciences and received cod no. IR.AJUMS.REC.1396.418. The permission to determine the prevalence of the lice infestation was allowed from the health unit in the schools and Education and Training, Office of the province. Moreover, informed consents designed by Ahvaz Jundishapur University of Medical Sciences were given to the students one day before and their parents’ signatures show their agreement that children took part in the project.

### Sample size & completing questionnaire 

The statistical community in this epidemiological study included girl primary school students, both urban and rural areas. The county has 30 girls' schools with 7963 students. The sample size was calculated by assuming an expected prevalence (p) of 11% with a 95% confidence and using the formula: n=pqz^2^ / d^2^. In which z=1.96, q=1- p=89% and d=0.2*p. Therefore, with 10% drop out rate, the sample size was computed as 855. The sample size was determined in each school proportional to the number of students.

Students were examined visually by combing the hair and directly interviewed to complete the questionnaire. Postgraduate medical entomology student was inspected the behind of the ears and the neck under hairs and scalp for five minutes^19^ to find nits, nymph, and adult of *P. capitis.*

### Statistical analyses

Results were presented as absolute frequencies and percentages for qualitative variables. First, univariate logistic regression models were used to examine whether there were relationships between the role of predictor variables and pediculosis status. The odds ratio (OR) was calculated and presented with a 95% confidence interval (95% CI). Any variable having a *P*-value less than 0.20 was selected as a candidate for the multiple logistic regression analysis. Backward stepwise logistic regression modeling was then used to obtain a subset of factors associated with pediculosis infection. Statistical analysis was performed using the statistical software SPSS 18.0.0. (SPSS Inc. Chicago, IL, USA). A *P*-value of less than 0.05 was considered significant.

## Results

About 851 students took part in this survey and 199 (23.38%) girls were at least infected with one of the growth stages of the parasite (nit, nymph and adult). The mean age of students was 9.42±1.68 (Infected: 8.81±1.54, Non-infected: 9.60±1.60) yr (Table 1).

**Table 1:** Prevalence of head lice with socio-demographic and other associated factors

**Variable**	**With p ediculosis**		**Without p ediculosis**		**Total**	
	**Number**	**Percent**	**Number**	**Percent**	**Number**	**Percent**
Location						
Urban	144	72.4	452	69.3	596	70.0
Rural	55	27.6	200	30.7	255	30.0
Number of students in class						
<30	73	39.7	236	39.7	309	39.7
≥30	111	60.3	357	60.3	468	60.3
Grade of education						
I–III	139	69.8	307	47.1	446	52.4
IV–VI	60	30.2	245	53.9	405	47.6
Father's Job						
Employed	138	72.6	532	85.4	670	82.4
Jobless	52	27.4	79	14.6	143	17.6
Mother's Job						
Employed	18	9.1	44	6.8	62	7.3
Housewife	179	90.9	603	93.2	782	92.7
Family size						
Low population (≤4)	48	24.1	175	26.8	223	26.2
Medium population (5-7)	130	65.3	416	63.8	546	64.2
Crowded	21	10.6	61	9.4	82	9.6
Shared personal hygiene products						
Common	82	41.2	168	25.8	250	29.4
Uncommon	117	58.8	484	74.2	601	70.6
Number of combing per day						
0-1	53	27.3	108	16.8	161	19.2
>1	141	72.7	535	83.2	676	80.8
Father's education						
Illiterate	18	9.7	41	6.6	59	7.4
Educated	167	90.3	574	93.4	741	92.6
Mother's education						
Illiterate	36	19.2	78	12.5	114	13.9
Educated	152	80.8	551	87.5	703	86.1
Number of bathing per week						
0-1	30	15.3	85	13.3	115	13.7
>1	166	84.7	557	86.7	723	86.3
Permanent head cover at home						
Negative	66	33.6	272	43.5	338	41.3
Positive	130	66.4	352	56.5	482	58.7
Hair type						
Straight	140	70.4	459	70.4	599	70.4
Curly	59	29.6	193	29.6	252	29.6
Using hair oil						
No	127	63.8	375	57.5	502	59.0
Yes	72	36.2	277	42.5	349	41.0
Dandruff						
Negative	141	70.9	455	69.8	596	70.0
Positive	58	29.1	197	30.2	255	30.0
Infected other family members						
Negative	164	84.2	623	97.2	787	94.2
Positive	31	15.8	18	2.8	49	5.8
Previous Infection/treatment						
Negative	59	30.3	425	70.3	484	60.5
Positive	136	69.7	180	29.7	316	39.5
Treatment type						
Traditional	75	65.3	101	64.4	176	64.7
Chemical	17	14.7	13	8.3	30	11.0
Both	23	20.0	43	27.3	66	24.3

Univariate logistic regression indicated that the prevalence of pediculosis was directly associated with grade, father’s job, shared personal hygiene products, number of combing, having a permanent head cover at home, infection in other members of the family and previous infection (Table 2).

**Table 2:** Univariate logistic regression predicting head lice in students

**Variables**	**OR (95% CI)**	***P*** **-value**
Location		
Urban	1.000	
Rural	0.863 (0.607, 1.228)	0.413
Number of students in class		
<30	1.000	
≥30	1.005 (0.717, 1.409)	0.967
Healthcare Teacher at school		
Positive	1.000	
Negative	1.161 (0.838, 1.609)	0.369
Grade of education		
I–III	1.000	
IV–VI	0.384 (0.274, 0.539)	0.001
Father's Job		0.001
Employed	1.000	
Jobless	2.203 (1.493, 3.250)	0.001
Mother's Job		
Housewife	1.000	
Employed	1.370 (0.777, 2.445)	0.273
Family size		0.701
Low population (≤4)	1.000	
Medium population (5-7)	1.139 (0.783, 1.658)	0.469
Crowded (≥8)	1.255 (0.696, 2.264)	0.450
Shared personal hygiene products		
Uncommon	1.000	
Common	2.019 (1.448, 2.815)	0.001
Number of combing per day		
0-1	1.000	
>1	0.462 (0.538, 1.325)	0.462
Father's education		
Educated	1.000	
Illiterate	1.509 (0.845, 2.696)	0.165
Mother's education		
Educated	1.000	
Illiterate	1.673 (1.084, 2.582)	0.020
Number of bathing per week		
0-1	1.000	
>1	0.462 (0.538, 1.325)	0.462
Permanent head cover at home		
Negative	1.000	
Positive	1.522 (1.088, 2.130)	0.014
Hair type		
Straight	1.000	
Curly	1.002 (0.708, 1.419)	0.990
Using hair oil		
No	1.000	
Yes	0.768 (0.553, 1.419)	0.114
Dandruff		
Negative	1.000	
Positive	0.950 (0.671, 1.346)	0.773
Infected other family members		
Negative	1.000	
Positive	6.542 (3.570, 11.990)	0.001
Previous Infection/treatment		
Negative	1.000	
Positive	5.443 (3.829, 7.737)	0.001
Treatment type		0.240
Traditional	1.000	
Chemical	1.760 (0.806, 3.847)	0.156
Both	1.475 (0.760, 2.861)	0.250

The descriptive study reported 72.4% of infected students lived in urban areas while 27.6% of them were in rural areas. About 60.3% of infected students were in crowded classes (30≤). Around 62.3% of individuals with head lice did not have health care teacher at the school, although 37.7% of them had associated teachers. The head lice infestation was observed in 65.3% of families with 5-7 members. Moreover, the father’s and mother’s jobs of 72.6% and 90.9% of the students with positive pediculosis were employed and housewife, respectively. There was a significant correlation between pediculosis infection and father’s job (OR: 2.203, 95% CI: 1.493, 3.250, *P*<0.0001).

*P. humanus capitis* was found in 69.8% of girls who were in the grade of I to III and 30.2% of them were in IV-VI grades. The grade was a significant variable against head lice (OR: 0.384, 95%CI: 0.274, 0.539, *P*<0.001). In infected groups, 41.2% used shared personal hygiene products. The prevalence of *P. humanus capitis* was directly associated with the common use of personal instruments (OR: 2.019, 95% CI: 1.448, 2.815, *P*<0.001). About 84.6% of positive students took a bath more than one time during a week. More than one-time combing was reported in 72.7% of infected groups. This survey declared a significant difference between the number of combing per day and pediculosis (OR: 0.537, 95% CI: 0.368, 0.783, *P*=0.001). 70.4% of infected students had straight hair and 29.6% of them had curly hair. However, 66.3% of infected schoolchildren had a permanent head cover at home. The head lice infestation had a significant association with the head cover (OR: 1.522, 95% CI: 1.088, 2.130, *P*=0.014).

We observed 70.9% of students with head lice did not have dandruff, and 68.3% of them had previous infection. Univariate logistic regression analysis showed a significant association between previous infection and *P. humanus capitis* (OR: 5.443, 95% CI: 3.829, 7.737, *P*<0.001). Furthermore, 36.2% of the patients used hair oil and 65.3% of them were used traditional treatments. In the case groups, 15.8% of family members were infected that it showed the relationship of head lice prevalence with family member infection (OR: 6.542, 95% CI: 3.570, 11.990, *P*<0.001).

Multivariate logistic regressions for predicting of head lice infection in girl students were reported in table 3. However, among 7 adjusted variables to the multivariate logistic regression model, grade (OR: 1.948, 95% CI: 1.307, 2.905, *P*=0.001), father’s job (OR: 2.385, 95% CI: 1.518, 3.750, *P*<0.001), shared personal hygiene products (OR: 1.817, 95% CI: 1.224, 2.698, *P*=0.003) and permanent head cover at home (OR: 1.399, 95% CI: 0.934, 2.097, *P*=0.104), using hair oil (OR: 1.904, 95% CI: 1.279, 2.836, *P*= 0.002). Students with jobless fathers were approximately 2.3 fold at risk of head lice infection. In girls with sharing personal instruments, it was observed 1.8 folds greater than the others.

**Table 3:** Multivariate logistic regression predicting pediculosis infection in students

**Variables**	**OR (95% CI)**	***P*** **-value**
Grade of education		
I–III	1.000	
IV–VI	1.948 (1.307, 2.905)	0.001
Father's Job		
Employed	1.000	
Jobless	2.385 (1.518, 3.750)	0.001
Shared personal hygiene products		
Uncommon	1.000	
Common	1.817 (1.224, 2.698)	0.003
Permanent head cover at home		
Negative	1.000	
Positive	1.399 (0.934, 2.097)	0.104
Using hair oil		
Yes	1.000	
No	1.904 (1.279, 2.836)	0.002
Infected other family members		
Negative	1.000	
Positive	3.1960 (1.597, 6.394)	0.001
Previous Infection/treatment		
Negative	1.000	
Positive	4.560 (3.075, 6.762)	0.001

## Discussion

A head louse is a serious and common public health problem among school children in the world^20^. In the current study, prevalence of pediculosis was directly associated with the grade of education, father’s job, shared personal hygiene products, number of combing, having permanent head cover at home, infection in other members of family and previous infection. The prevalence of head lice was detected 23.38% among the girl students in Karoon County. Other epidemiological studies conducted among children of elementary schools in EMRO countries reported that the prevalence of head lice was 13.1% in Kayseri City of Turkey^21^, 33% in Sharkia Governorate of Egypt^22^, 45.45% in Albaha Region Kingdom of Saudi Arabia^23^, 14.3% in Damascus City of Syria^24^.

In a meta-analysis and systematic review study, the lowest and the highest prevalence of *P. capitis* infestation were 0.47% and 27% in the center and southeast of Iran^13^ but the most infestation (67.3%) were reported from Bashagard County in Bandarabbas Province^25^. The head lice infestation during 2010 to 2014 was reported 25.7% (Feb), 22.7% (Dec), 15.4% (Mar), 22.4% (Dec) and 25.2% (Mar) from Ahvaz, respectively^26^. The high head lice prevalence in present survey is likely due to absent educational hygiene programs, poor knowledge and disability in early detection of the ectoparasite and also, cultural, economic and social factors in Karoon County. In addition, the increase in the head lice prevalence has a direct relation to climatic condition ^27, 28^. Therefore, the previous study showed high infestation in tropical areas^29^.

*P. capitis* frequency was higher than in rural areas^21^. The present study revealed the infestation in urban areas is higher than in rural areas. The difference is due to sampling and existence of more schools in urban areas than rural areas. Health care teachers have an effective role in the reduction of the head lice infestation and rising health awareness of students. This finding was observed in our study and supported by previous research^30^.

Our results showed the head lice infestation was correlated with the grade of education, head cover, the length of hair, family history of infestation, agreed to other studies^30-32^.

In this study, there was no significant relationship between the mother's job and lice infestation. Mothers who work outside do not likely have the opportunity to look after their children and their housewife are not able to control and treat the head lice infestation due to their low awareness^3^. Moreover, mothers employed have to leave the children in the nursery or kindergarten during the day that it may raise the risk of head lice transmission by the close child to child contact^33^. Shared personal hygiene products were directly related to the prevalence of *P. humanus capitis* ^31, 34^. Our result was completely according to previous researches.

Our results demonstrated that using hair oil, the presence of dandruff on the head and treatment type in the previous infestation was not related to the prevalence of *P. humanus capitis*. There was no significant difference between infested and non-infested children in view of the presence of dandruff^35^. Moreover, there was no significant correlation between the family size and prevalence of head lice in our survey. This finding can confirm the likely head lice transmission between students in schools and in the socioeconomic low-level family^36^.

The present study showed that girls aged 7-9 yr presented a higher head lice infestation in compare with ones aged 10-12 year. Similar results have been reported from other countries including India ^37^and Pakistan ^38^. This may be due to closer contact between girls aged 7-9 yr groups than older ones.

Students whose fathers were jobless were almost twice more be infested compared with other groups. This finding was supported by the study was done in the south of Iran^39^. The reason can explain by poor hygiene and economic status^36^. Poverty is directly related to head lice infestation^33^.

The cross-sectional study has weakness in the detection of causation, and temporality is the limitation of the project. Other potential limitations are ignoring the boy’s elementary schools due to ethical issue and private schools regarding lack of admission to schools.

## Conclusion

In regards to the high prevalence of head lice in the present study, the health center of Karoon County needs to change its policy for parasite control and treatment. Health authorities should encourage families to increase their awareness about identifying head lice growth stages (nit, nymph, and adult) and prevention and treatment methods of the ectoparasite. Furthermore, health education programs are necessary to increase knowledge of the students, teachers and their families. Moreover, the education and Training Office must monitor the infestation prevalence among students in Karoon County.

## Acknowledgements

The authors would like to thanks to the Education and Training Office for good cooperation. This article is part of Hoda Ghofleh Maremazi’s MSPH thesis, financially supported by Ahvaz Jundishapur University of Medical Sciences (AJUMS). (Project No. u-96076).

## Conflict of interest

The authors declare that there is no conflict of interests.

## Funding

The project is funded by Ahvaz Jundishapur University of Medical Sciences.

## Highlights

- Head lice infestation was estimated 23.38% among girl’s elementary schools.
- Students with jobless fathers were 2.3 folds at risk of head lice infection.
- The permanent head cover at home did not predict the prevalence of head lice.
